# Clinical relevance of the interleukin 10 promoter polymorphisms in Chinese Han patients with major trauma: genetic association studies

**DOI:** 10.1186/cc8182

**Published:** 2009-11-26

**Authors:** Ling Zeng, Wei Gu, Kehong Chen, Dongpo Jiang, Lianyang Zhang, Dingyuan Du, Ping Hu, Qing Liu, Suna Huang, Jianxin Jiang

**Affiliations:** 1 State Key Laboratory of Trauma, Burns and Combined Injury, Research Institute of Surgery, Daping Hospital, Third Military Medical University, Changjiang Road, Yuzhong District, Chongqing, 400042, PR China; 2 Department of Traumatic Surgery, Daping Hospital, Third Military Medical University, Changjiang Road, Yuzhong District, Chongqing, 400042, PR China; 3 Chongqing Emergency Medical Center, Jiankang Road, Yuzhong District, Chongqing, 400042, PR China

## Abstract

**Introduction:**

An excessive inflammatory response is thought to account for the pathogenesis of sepsis and multiple organ dysfunction syndrome (MODS) after severe trauma. The interleukin-10 (IL-10) is a potent anti-inflammatory cytokine. The objectives of this prospective study were to investigate the distribution of IL-10 promoter polymorphisms in a cohort of 308 Chinese Han patients with major trauma, and to identify associations of IL-10 promoter polymorphisms with IL-10 production and incidence of sepsis and MODS.

**Methods:**

A total of 308 patients with major trauma were included in this study. The genotypes of polymorphisms -1082, -819 and -592 were determined by polymerase chain reaction-restriction fragment length polymorphism. The IL-10 levels in the supernatants were determined with enzyme-linked immunoabsorbent assay.

**Results:**

The -1082A and -592A alleles were significantly associated with lower lipopolysaccharide-induced IL-10 production in an allele-dose dependent fashion. There was no significant difference for the -819 polymorphism. Except for the -1082 polymorphism, the -819 and -592 polymorphisms were not significantly associated with sepsis morbidity rate and MOD scores.

**Conclusions:**

Our results further confirm the functionality of the IL-10 promoter single nucleotide polymorphisms in relation to IL-10 production. They also suggest that individual difference in IL-10 production in trauma patients might be at least in part related to genetic variations in the IL-10 promoter region.

## Introduction

Trauma, a major public health problem worldwide, ranks as the fourth leading cause of death among all diseases [1]. One of the most serious complications of major trauma is the sequential dysfunction of vital organs, which is associated with post-traumatic sepsis in the majority of cases [2]. Therefore, preventing sepsis and subsequent organ dysfunction is crucial in the treatment of surviving patients with major trauma. It has been demonstrated that inappropriate immune inflammatory response contributes to the development of sepsis and multiple organ dysfunction syndrome (MODS) in major trauma patients [3,4]. Increasing evidence suggests that genetic variants, particularly single nucleotide polymorphisms (SNPs), are critical determinants for inter-individual differences in both inflammatory responses and clinical outcome in trauma patients [5,6]. Delineating the variation in genes and associated differences in response to trauma may contribute to the development of new genetically tailored diagnostic and therapeutic interventions that will improve outcome in the patients with major trauma.

IL-10 is one of the key anti-inflammatory cytokines and it decreases the production of inflammatory molecules, such as TNF-α, interferon (IFN)-γ, IL-12, reactive nitric oxide metabolites, major histocompatibility complex molecules [7] and inhibits antigen-specific cytotoxic T cells [8]. The magnitude of the proinflammatory cytokine response, neutrophil infiltration, and injury were shown to be greater in IL-10^(-/-)^-null mice after visceral ischemia-reperfusion injury. Administration of exogenous IL-10 results in a decrease in neutrophil infiltration in IL-10^(-/-)^-null mice [9]. It suggests that lower producers of IL-10 might be sensitive to the development of sepsis and MODS due to its ability to suppress inflammation. IL-10 has been shown to be elevated after trauma [10] and is well correlated with the development of sepsis and outcome in patients with major trauma [11,12]. Previous studies have demonstrated that the variation in IL-10 production is largely genetically determined, which is mainly related to genetic variations in the promoter region [13,14]. The IL-10 5'-flanking region, which controls transcription, is polymorphic, with two microsatellites between -4000 and -1100, and three SNPs (-1082, -819, and -592) [15]. The -1082, -819 and -592 polymorphisms are shown to be in close linkage disequilibrium and construct only three haplotypes in the white population (GCC, ACC and ATA in an order of -1089/-819/-592) [16]. These haplotypes are associated with high (GCC), intermediate (ACC), and low (ATA) IL-10 production [14]. Genetic association studies have indicated that the three SNPs in the IL-10 promoter are linked to various diseases, such as Crohn's disease [16], schizophrenia [17], hepatitis [18], endometriosis [19], Alzheimer's disease [20], acute respiratory distress syndrome [21] and sepsis [22]. However, clinical relevance of the three SNPs is still not fully understood, due to considerable controversy in the literature regarding the influence of these SNPs on susceptibility to diseases and their functionality [23-25]. Schroeder and colleagues [23,26] reported the association of the IL-10 with the -1082 and -592 polymorphisms with the incidence of MODS and acute respiratory failure in patients with major trauma, respectively, and also showed inconsistent results.

In this study we investigated whether the genetic variations at positions -1082, -819 and -592 in the IL-10 promoter affect IL-10 production after trauma, and whether there is an association of these polymorphisms with the development of post-traumatic sepsis and MODS. Our hypothesis was that genetically determined lower production of IL-10 might increase susceptibility to post-traumatic complications.

## Materials and methods

### Study population

A total of 308 patients with major trauma (240 male and 68 female) were prospectively recruited in this study. All of them are Han Chinese and live in Chongqing district. The patients were consecutively admitted to the Department of Trauma Surgery in the Daping Hospital and the Chongqing Emergency Medical Center between 1 January, 2005 and 1 June, 2008. They were enrolled in the study if they met the following criteria: (1) aged between 18 and 65 years; (2) expected Injury Severity Score (ISS) greater than 16 combined with the presence of at least one life-threatening injury and at least one additional severe injury in another part of the body and (3) probability of survival greater than 48 hours. Patients were not eligible if they had penetrating injuries, or pre-existing cardiovascular, respiratory, renal, hepatic, hematologic or immunological diseases. ISS was performed according to the Abbreviated Injury Scale 2005 by independent evaluators [27]. All patients requiring surgical intervention received standard surgical care and postoperative intensive care unit treatment. The protocol for this study was approved by the Ethical and Protocol Review Committee of the Third Military Medical University, and informed consent was obtained from the patients or the patient's next of kin.

### Genotyping of the IL-10 promoter polymorphisms

Blood specimens were collected in tripotassium ethylenediamine tetraacetic acid sterile tubes from trauma patients immediately after admission (generally within 10 hours after injury) in order to avoid the effect of blood transfusion. The genomic DNA was isolated from whole blood using Wizard genomic DNA purification kit (Promega, Madison, WI, USA) according to the manufacturer's protocol. A PCR-restriction fragment length polymorphism (RFLP) method was used to detect the IL-10 promoter polymorphisms. The oligonucleotide primers for amplification and PCR conditions were shown in Table 1. PCR products were digested with XagI, Hin1α or Rsa I (New England Biolabs, Beverly, MA, USA) for one hours at 37°C. The SNPs were then genotyped by fragment size obtained after agarose gel electrophoresis, which were further confirmed by DNA sequencing of the IL-10 promoter with 10 random samples (Takara Biotech, Dalian, China). The genotyping was performed in a blinded fashion such that those analyzing the genotype data did not know any other experimental results.

**Table 1:** Primers and endonucleases for genotyping of IL-10 promoter single nucleotide polymorphisms

SNPs	Primers	PCR conditions	Restriction endonucleases
-1082A/G	F:ACACAAATCCAAGACAACACTACTAAGGCTTCCTTGGGA	3 minutes at 94°C followed by 35 cycles of 40 seconds at 94°C, 45 seconds at 56°C, 40 seconds at 72°C, then 10 minutes at 72°C	XagI
	R:GTGATCAAACAGAGGCACAGACAT		
-819T/C	F:CACTCTAAGGCTTCCTTGGGA	3 minutes at 94°C followed by 35 cycles of 40 seconds at 94°C, 45 seconds at 56°C, 40 seconds at 72°C, then 10 minutes at 72°C	Hin1α
	R:CCTACCGTCTCTATTTTATAGTGAGCAAACTGAGGCACAGACAT		
-592 A/C	F:AGCTGAAGAGGTGGAAACATGTG	3 minutes at 94°C followed by 35 cycles of 40 seconds at 94°C, 45 seconds at 63°C, 40 seconds at 72°C, then 10 minutes at 72°C	Rsa I
	R:TGGGTTCTCATTCGCGTGTT		

### *Ex vivo *IL-10 production

A human whole-blood assay was used as described previously [6]. In brief, aliquots of whole blood collected from the trauma patients were mixed in a ratio of 1:1 with Roosevelt Park Memorial Institute medium 1640, and incubated with 100 ng/ml lipopolysaccharide (LPS; *Escherichia coli *O26:B6, Difco Laboratories, Detroit, MI, USA) in a sample mixer at 37°C for four hours. The supernatants were then separated by centrifugation. The IL-10 levels in the supernatants were determined by ELISA according to the manufacturer's instructions (R & D System, Minneapolis, MN, USA). The lower limit for detection was 4 pg/ml. Variability between triplicate wells was less than 5%.

### Clinical evaluation

After admission, the patients with major trauma were monitored in the following aspects: respiratory (partial pressure of arterial oxygen/fraction of inspired oxygen ratio), renal (serum creatinine concentration), hepatic (serum bilirubin concentration), cardiovascular (pressure-adjusted heart rate) and hematologic (platelet count) systems. The organ function was then scored using the method of Marshall and colleagues [28] and calculated as a single daily value during the intensive care unit stay. Neurological scoring was not performed because every patient was sedated. Sepsis was defined if patients met all the following criteria: clinical evidence of infection, body temperature greater than 38.5°C or less than 36.5°C, and leukocyte count greater than 10 × 10^9^/L or less than 4 × 10^9^/L. The MODS scores and sepsis were determined by individuals who did not know the genotypes.

### Statistical analysis

Sample size was calculated using online Power and Sample Size Program software [29]. The desired power of our study was set at 80% with a significance level of 0.05 in a two-sided test. We chose the log-additive inheritance model, which is the most suitable for polygenic diseases. By means of the Power and Sample Size program, our sample (n = 308) was considered adequate to study the -1082, -819 and -592 polymorphisms.

Allele frequencies for each SNP were determined by gene counting. Any deviation from Hardy-Weinberg equilibrium was calculated by a chi-squared goodness-of-fit test. The chi-squared test or Fisher's exact test was used to determine differences in frequencies of the IL-10 promoter polymorphisms among trauma patients with different genotypes. Odds ratios (OR) and 95% confidence intervals (CI) were calculated using logistic regression models whenever that chi-squared or Fisher's exact test was significant. The association of the IL-10 promoter polymorphisms with plasma IL-10 levels and MODS scores was determined using one-way analysis of variance. Three genetic models (allele-dose, dominant, and recessive) were used. Significant probability values obtained were corrected for multiple testing (Bonferroni correction). *P *values less than 0.05 were considered significant. All statistic analysis was carried out using SPSS Version 11.0 (SPSS Inc, Chicago, Illinois, USA).

## Results

### Overall clinical characteristics of patients with major trauma

The patient cohort comprised a total of 308 consecutive Han Chinese patients with severe multiple trauma. Trauma was caused by traffic accidents (n = 187), falling (n = 105), or other causes (n = 16). Baseline data of the patients are shown in Table 2. The severely injured patients (mean ISS 25.5 ± 8.2) were mostly young (38.5 ± 11.5 years). One hundred and forty seven patients (47.7%) developed sepsis. Two hundred and fifty five (82.8%) patients developed organ dysfunction, among whom one hundred and sixty (52%) had two or more organ dysfunctions. The mean time to MODS was 4.4 ± 3.5 days. The mean number of operations performed per patient was 3.5 ± 3.2 (range 1 to 14). Substitution of erythrocytes was necessary in every patient (mean 5.4 ± 5.2 L). All of the patients survived at least 48 hours after admission and completed genotyping.

**Table 2:** Overall clinical characteristics of patients with major trauma (n = 308)

Age (years)	38.5 ± 11.5 (18-65)
Male/female, n	240/68
ISS	25.5 ± 8.2
≥ 16, <25, n	143
≥ 25, n	165
Injured body regions	
Head, n (AIS points)	115 (1.9 ± 1.7)
Thorax, n (AIS points)	123 (3.3 ± 1.3)
Abdomen, n (AIS points)	97 (3.0 ± 1.9)
Extremities, n(AIS points)	165 (2.2 ± 1.1)
Number of regions injured	
Two, n	186
Three, n	99
All four, n	23
Organ dysfunction	
One, n	95
Two, n	101
Three or above, n	59
Sepsis, n (%)	147(47.7)

AIS = Abbreviated Injury Scale; ISS = Injury Severity Scores.

### Allele frequencies and genotype distribution

The overall minor allele frequencies were 15.4%, 29.2% and 34% for the -1082G, -819C and -592C alleles, respectively, in our cohort. The genotype frequencies of these three SNPs were in agreement with the Hardy-Weinberg equilibrium (Table 3). These SNPs were completely (-819 to -592) or strongly (-1082 to -819 and -592) linked. The three common haplotypes with frequency of more than or equal to 10% were -1082A/-819T/-592A (ATA, 49.5%), -1082A/-819T/-592C (ATC, 14.1%) and -1082A/-819C/-592A (ACA, 13.1%), respectively. With regard to the number of ATA haplotypes, 25.7% of the trauma patients had the genotype 0 ATA, 51.2% had 1 ATA, and 23.1% had 2 ATA.

**Table 3:** Distribution of the IL-10 promoter polymorphisms among trauma patients

Loci	Patients	n	MA (%)	Genotypes, n (%)			HWE
					
				wild	heterozygous	variant	
A-1082G	All	308	15.4	224 (72.7)	73 (23.7)	11 (3.6)	0.11
	Sepsis	147	12.2	115 (78.2)	28 (19.1)	4 (2.7)	
	Non-sepsis	161	18.3^a^	109 (67.7)^b^	45 (28)	7 (4.3)	
T-819C	All	308	29.2	157 (51.0)	122 (39.6)	29 (9.4)	0.46
	Sepsis	147	29.3	73 (49.7)	62 (42.2)	12 (8.2)	
	Non-sepsis	161	29.2	84 (52.2)	60 (37.3)	17 (10.6)	
A-592C	All	307	34.0	141 (45.9)	123 (40.1)	43 (14.0)	0.07
	Sepsis	147	34.4	65 (44.2)	63 (42.9)	19 (12.9)	
	Non-sepsis	160	33.8	76 (47.5)	60 (37.5)	24 (15.0)	

Sepsis vs. non-sepsis: ^a^*P *= 0.037, ^b^*P *= 0.038. HWE = Hardy-Weinberg equilibrium; MA = minor allele.

### Association of the IL-10 promoter polymorphisms with IL-10 production

Given the possible functionality of the -1082, -819 and -592 polymorphisms, we hypothesized that these SNPs might affect IL-10 production after trauma, leading to individual difference in plasma IL-10 levels in trauma patients. Peripheral blood was taken immediately after admission in an attempt to avoid the potential effects of non-genetic factors on IL-10 production. As shown in Table 4, there were no significant differences in age, gender ratio and ISS scores among trauma patients with different genotypes of the three polymorphisms. Spontaneous IL-10 production was not significantly different between different genotypes of all three loci (data not shown). However, LPS-induced IL-10 production was shown to be significantly different between different genotypes of the -1082 and -592 polymorphisms, showing that the -1082A and -592A alleles were associated with lower IL-10 production (*P *= 0.005 and *P *= 0.001, respectively by dominant effect). Data from linear regression analysis indicated that the functional association of these two polymorphisms with IL-10 production was significantly allele-dose dependent (*P *= 0.003 and *P *= 0.037 for -1082 and -592, respectively). Although the -819T allele is also associated with lower LPS-induced IL-10 production, no significant difference was found between the different genotype groups (Table 4). In case of a combination effect of the three SNPs, we further analyzed the association of ATA haplotype with IL-10 production. Table 5 showed that the production of IL-10 was lowest in those with haplotype genotype 2 ATA, showing significant difference if compared with those with genotype 0 ATA (*P *= 0.041). Data from linear regression analysis indicated that this association with IL-10 production were significantly haplotype-dose dependent (*P *= 0.041).

**Table 4:** Clinical relevance of the IL-10 promoter polymorphisms in patients with major trauma

Genotypes	N	Age (years)	Gender (M/F)	ISS	Sepsis(%)	MOD score	Cytokine (pg/ml)
-1082							
AA	224	38.6 ± 14.1	177/47	25.6 ± 8.5	51.3	4.4 ± 2.7	62.0 ± 19.8
AG	73	37.3 ± 13.5	62/11	26.3 ± 7.5	38.4	5.4 ± 2.8	73.9 ± 21.3
GG	11	35.9 ± 20.3	7/4	27.2 ± 10.6	36.4	4.1 ± 2.9	86.0 ± 18.8
-819					a1	b1	a2, b2, c1
TT	157	37.6 ± 13.7	120/37	25.6 ± 8.8	46.5	4.6 ± 2.6	56.2 ± 17.8
TC	122	39.6 ± 14.6	100/22	25.9 ± 8.1	50.8	4.7 ± 2.8	59.9 ± 20.2
CC	29	35.7 ± 15.1	26/3	27.1 ± 7.9	41.4	4.4 ± 2.9	62.4 ± 19.5
-592							
AA	141	38.2 ± 14.6	116/25	25.1 ± 7.9	46.1	4.5 ± 2.1	52.9 ± 16.0
AC	123	38.0 ± 13.2	98/25	27.1 ± 9.0	51.2	5.1 ± 3.0	66.8 ± 19.6
CC	43	36.8 ± 16.0	31/12	24.8 ± 8.0	44.2	4.1 ± 2.7	59.8 ± 13.1
							a3, c2

a: dominant effect (variant homozygotes +heterozygotes vs. wild homozygotes) as analyzed by ANCOVA, ^a1^*P *= 0.038, ^a2^*P *= 0.005, ^a3^*P *= 0.001.

b: recessive effect (variant homozygotes vs. heterozygotes + wild homozygotes) as analyzed by ANCOVA, ^b1^*P *= 0.088, ^b2^*P *= 0.083.

c: allele dose effect as analyzed by linear regression analysis, ^c1^*P *= 0.003, ^c2^*P *= 0.037.

ANCOVA = analysis of covariance; F = female; ISS = Injury Severity Score; M = male; MOD = multiple organ dysfunction.

**Table 5:** Clinical relevance of Interleukin-10 haplotypes in major trauma patients

Haplotype*	Count	IL-10 (pg/ml)	MODS	Sepsis (%)
0(ATA)	71	72.18 ± 22.51	4.25 ± 2.60	28 (39.4)
1(ATA)	157	65.19 ± 20.36	5.00 ± 2.89	76 (48.4)
2(ATA)	79	60.27 ± 19.24^a, c^	4.24 ± 2.39	43 (54.4)

*'0 ATA haplotype' includes the following genotypes: ATC/ATC, ATC/ACC, ACA/ACA, ACA/ACC, ACC/ACC, ATC/GTC, ATC/GCC, ACC/GTC, ACA/GCA, ACC/GCA, ACA/GCC, ACC/GCC, GTA/GTC, GTC/GTC, GTA/GCC and GTC/GCA; '1 ATA haplotype' includes ATA/ATC, ATA/ACA, ATA/ACC, ATA/GTA, ATA/GTC, ATA/GCA and ATA/GCC; '2 ATA haplotype' is ATA/ATA. haplotype is indicated in an order of -1082A, -819T and -592A.

a: 2 ATA vs 0 ATA, *P *= 0.041; c: haplotype dose effect, *P *= 0.041.

MODS = multiple-organ dysfunction syndrome.

### Association of the IL-10 promoter polymorphisms with development of sepsis and MODS in trauma patients

As shown in Table 4, there was a close association of the -1082 polymorphism with the development of sepsis, showing that the patients with the A allele had significantly higher sepsis morbidity (*P *= 0.038 for dominant effect). Data from multiple logistical regression analyses indicated that A to G variation at this position was borderline significantly associated with lower risk of sepsis (OR = 0.677, 95% CI = 0.453 to 1.011, *P *= 0.057). In addition, there was a borderline significantly increasing trend of MODS scores in the patients with the A allele at -1082 locus (*P *= 0.088 for recessive effect). However, both of the -819 and -592 polymorphisms were not shown to be significantly associated with the development of sepsis and MODS in trauma patients.

Concerning the association of sepsis with allele and genotype frequencies, there were no significant differences in allele and genotype frequencies of the three polymorphic loci between all trauma patients and trauma patients with sepsis. There was a significant difference in the AA genotype frequency (*P *= 0.038) at position -1082 between patients with and without sepsis. The frequency of the G allele was significantly higher in patients without sepsis than in those with sepsis (*P *= 0.037). No significant difference was observed between patients with and without sepsis for the allele and genotype frequencies of other two polymorphic loci (Table 3). With respect to the combination effect of the three SNPs, although there was a trend of increasing risk for sepsis incidence with the number of ATA haplotype (39.4% for 0 ATA vs 54.4% for 2 ATA), no significant differences in MODS scores and sepsis morbidity rate were observed between any haplotype groups (Table 5).

## Discussion

The aim of the present study was to estimate the clinical relevance of the IL-10 promoter polymorphisms in patients with major trauma. The described genotype and allele frequencies of the IL-10 polymorphisms studied in this cohort are quite similar to previously reported distributions in other studies in Chinese patients [18-20,25] and other Asian populations [30]. The minor alleles at positions -819 and -592 are the C allele in Asian populations [18-20,25,30], which is different to those seen in Western populations, being T and A alleles, respectively [22-24,26,31]. Although the minor allele at position -1082 is G in both Asian and Western populations, its frequency is quite different, over 40% in Western populations [21,22,26,31,32] and about 10% in Asian populations [19-21]. This leads to quite a difference in the common haplotypes between Asian and Western populations. Three common haplotypes in this cohort are ATA, ATC and ACA, respectively, while GCC, ATA and ACC are the common haplotypes in Western populations. The differences in allele prevalence might be due to ethnic difference [33].

It has been shown that about 75% of the variation in IL-10 production is genetically determined [13]. IL-10 production appears to be controlled at the transcriptional level [34]. The -1082, -819 and -592 polymorphisms have been shown to affect promoter activity, showing that the ATA haplotype is associated with the lowest transcription activity [19]. Results from bioinformatics analysis indicates that base pair substitutions at positions -1082, -819 and -592 might affect putative transcription factor binding, such as C/EBP beta, GATA-binding factor 2, Ets and STAT3 [35]. Given the functionality of the IL-10 promoter SNPs, we investigate whether these SNPs affect IL-10 production in response to a known stimulus such as LPS immediately after trauma. In the case of the effect of individual SNP, our results indicate that both of the -1082 A and -592 A alleles are shown to be associated with LPS-induced IL-10 production. However, there is no association for the -819 polymorphism. Our results are in agreement with previous reports [15,34], but in contrast to the results reported by Keijsers and colleagues [36] and Rad and colleagues [37]. They showed that the A allele at -1082 and the T allele at -819 were associated with higher and lower IL-10 production, respectively. Similar to their effect on transcription, LPS-stimulating production of IL-10 by peripheral blood leukocytes is lowest in patients with combined genotype 2 ATA, which is significantly different compared with patients with genotype 0 ATA. The ATA haplotype has been demonstrated to have lowest transcriptional activity [19]. Our results further confirm the functionality of the IL-10 promoter SNPs in relation to IL-10 production. It also suggests that individual differences in IL-10 production in trauma patients might be at least in part related to genetic variations in the IL-10 promoter region.

Given the functional significance of the -1082 and -592 polymorphisms and ATA haplotype, we further hypothesized that the IL-10 promoter polymorphisms would be important in influencing severity to the development of sepsis and MODS in trauma patients by changing the balance of pro-inflammatory and anti-inflammatory cytokines. There were no significant differences in age, gender ratio and ISS scores among the different genotype groups, which minimized the influence of interfering factors. The patient cohort consisted of young patients with a low background of coexisting morbidity. Similar to their association with IL-10 production, genetic variations at the three loci have a trend to be associated with lower risk of sepsis and MODS. However, only the -1082 polymorphism has statistical significance, showing that the patients with the A allele had significantly higher sepsis morbidity and a borderline significant increase in MODS scores. There are also significant differences in the frequencies of the allele and AA genotype at this polymorphic locus between patients with sepsis and non-sepsis. The finding of no significant difference in the GG genotype frequency might be due to a small number of patients carrying the GG genotype in the current study. Results from Stanilova and colleagues [22] also revealed that the AA genotype of the -1082 locus was associated with lower IL-10 production in LPS-, phytohemagglutinin- or pokeweed mitogen-stimulated healthy peripheral blood leukocytes. Patients with severe sepsis were shown to have a significant elevation of A allele frequency when compared with healthy controls. In addition, Gong and colleagues [21] further reported that the high IL-10-producing -1082 GG genotype was protective against organ failure and mortality in acute respiratory distress syndrome. It is increasingly recognized that the overall balance between pro-inflammatory and anti-inflammatory responses is important in the development of sepsis and MODS after trauma. Therefore, it is reasonable that lower IL-10-producing -1082 AA genotype might be used as a relevant risk estimate for organ dysfunction and sepsis in trauma patients. This is not consistent with the report by Schroeder and colleagues, which showed that the -592 polymorphism, but not -1082, was associated with MODS in patients with major trauma [23]. The different patient populations studied may explain this discrepancy. With respect to the combination effect, our results show that the patients with 2 ATA haplotypes have higher sepsis morbidity rates, but this lacks statistical significance, although it is significantly associated with lower IL-10 production. This is in accordance with previous reports [38]. It suggests that there is a lack of synergistic effect among the -1082, -819 and -592 polymorphisms in relation to the development of sepsis and MODS in trauma patients. Other pathogenic factors should be also considered when explaining the current results. One important factor is the polygenetic and multifactorial involvement in the pathogenesis of sepsis and multiple organ dysfunction after trauma [39]. In fact, there is increasing evidence indicating that genetic polymorphisms of other genes are associated with the occurrence of post-traumatic complications, such as IL-1β, TNFα, heat shock protein 70, IFN-γ and IL-18 [40,41]. The susceptibility to sepsis and organ dysfunction in trauma patients might be the result of a combination of numerous genetic polymorphisms. In addition, the relatively small sample size we recruited in this study might also affect our conclusions. Our sample, although being considered adequate by means of the Power and Sample Size program, just has a size of about 300 patients, among which only 147 and 160 patients had sepsis and MODS, respectively. Further studies are needed to confirm the clinical relevance of the IL-10 promoter polymorphisms in a larger cohort of trauma patients and to investigate the relation of these SNPs with other genetic polymorphisms in prediction of sepsis and outcome in trauma patients.

## Conclusions

The present study investigated the clinical relevance of the genetic variations at positions -1082, -819 and -592 in the IL-10 promoter in patients with major trauma. These genetic variations are shown to affect IL-10 production after trauma, and to be associated with risk for the development of post-traumatic sepsis and MODS at different degrees. Further studies, both clinical and experimental, are therefore needed to confirm the significance of these findings and to investigate their synergistic effect with other genetic polymorphisms in relation to the development of sepsis and outcome in trauma patients.

## Key messages

• The -1082A and -592A alleles and ATA haplotype were significantly associated with lower IL-10 production in response to *ex vivo *LPS stimulation.

• The -1082 polymorphism was closely associated with the development of sepsis, showing that the patients with the A allele had significantly higher sepsis morbidity.

• There are no marked synergistic effects among the -1082, -819 and -592 polymorphisms in relation to the development of sepsis and MODS in trauma patients.

## Abbreviations

CI: confidence interval; ELISA: enzyme-linked immunosorbent assay; IFN-γ: interferon-γ; IL-10: interleukin-10; ISS: Injury Severity Score; LPS: lipopolysaccharide; MODS: multiple organ dysfunction syndrome; OR: odds ratios; PCR: polymerase chain reaction; RFLP: restriction fragment length polymorphism; SNPs: single nucleotide polymorphisms; TNF-α: tumor necrosis factor alpha.

## Competing interests

The authors declare that they have no competing interests.

## Authors' contributions

LZ and WG were the main researchers for this study and co-contributed to write this manuscript. K-HC, D-PJ, L-YZ, D-YD and PH were involved in the collecting of blood samples and clinical data. QL and S-NH did the technical work. J-XJ planned the study, wrote the protocol and was involved in the genetic and clinical aspects of data analyses and revised the manuscript. All authors read and approved the final manuscript.
